# Early Signals of Vaccine-driven Perturbation Seen in Pneumococcal Carriage Population Genomic Data

**DOI:** 10.1093/cid/ciz404

**Published:** 2019-05-16

**Authors:** Chrispin Chaguza, Ellen Heinsbroek, Rebecca A Gladstone, Terence Tafatatha, Maaike Alaerts, Chikondi Peno, Jennifer E Cornick, Patrick Musicha, Naor Bar-Zeev, Arox Kamng’ona, Aras Kadioglu, Lesley McGee, William P Hanage, Robert F Breiman, Robert S Heyderman, Neil French, Dean B Everett, Stephen D Bentley

**Affiliations:** 1 Parasites and Microbes Programme, Wellcome Sanger Institute, Wellcome Genome Campus, Cambridge; 2 Department of Clinical Infection, Microbiology and Immunology, Institute of Infection and Global Health, University of Liverpool, United Kingdom; 3 Malawi-Liverpool-Wellcome Trust Clinical Research Programme, Blantyre; 4 Darwin College, University of Cambridge, Silver Street, Cambridge; 5 HIV and STI Department, National Infection Service, Public Health England, London, United Kingdom; 6 Malawi Epidemiology Intervention Research Unit (formerly KPS), Chilumba; 7 Center of Medical Genetics, University of Antwerp, Belgium; 8 Medical Research Council Centre for Inflammation Research, Queens Medical Research Institute, University of Edinburgh, United Kingdom; 9 Mahidol Oxford Tropical Medicine Research Unit, Mahidol University, Bangkok, Thailand; 10 Nuffield Department of Medicine, University of Oxford, United Kingdom; 11 Department of International Health, Johns Hopkins Bloomberg School of Public Health, Baltimore, Maryland; 12 Department of Biomedical Sciences, University of Malawi, College of Medicine, Blantyre; 13 Respiratory Diseases Branch, Centers for Disease Control and Prevention, Atlanta, Georgia; 14 Center for Communicable Disease Dynamics, Department of Epidemiology, Harvard T. H. Chan School of Public Health, Boston, Massachusetts; 15 Hubert Department of Global Health, Rollins School of Public Health, Emory University, Atlanta, Georgia; 16 Division of Infection and Immunity, University College London, United Kingdom; 17 Department of Pathology, University of Cambridge, United Kingdom

**Keywords:** pneumococcus, carriage, genomics, serotypes

## Abstract

**Background:**

Pneumococcal conjugate vaccines (PCVs) have reduced pneumococcal diseases globally. Pneumococcal genomic surveys elucidate PCV effects on population structure but are rarely conducted in low-income settings despite the high disease burden.

**Methods:**

We undertook whole-genome sequencing (WGS) of 660 pneumococcal isolates collected through surveys from healthy carriers 2 years from 13-valent PCV (PCV13) introduction and 1 year after rollout in northern Malawi. We investigated changes in population structure, within-lineage serotype dynamics, serotype diversity, and frequency of antibiotic resistance (ABR) and accessory genes.

**Results:**

In children <5 years of age, frequency and diversity of vaccine serotypes (VTs) decreased significantly post-PCV, but no significant changes occurred in persons ≥5 years of age. Clearance of VT serotypes was consistent across different genetic backgrounds (lineages). There was an increase of nonvaccine serotypes (NVTs)—namely 7C, 15B/C, and 23A—in children <5 years of age, but 28F increased in both age groups. While carriage rates have been recently shown to remain stable post-PCV due to replacement serotypes, there was no change in diversity of NVTs. Additionally, frequency of intermediate-penicillin-resistant lineages decreased post-PCV. Although frequency of ABR genes remained stable, other accessory genes, especially those associated with mobile genetic element and bacteriocins, showed changes in frequency post-PCV.

**Conclusions:**

We demonstrate evidence of significant population restructuring post-PCV driven by decreasing frequency of vaccine serotypes and increasing frequency of few NVTs mainly in children under 5. Continued surveillance with WGS remains crucial to fully understand dynamics of the residual VTs and replacement NVT serotypes post-PCV.

## Teaser

After 13-valent pneumococcal conjugate vaccine (PCV13) introduction in Malawi, the frequency of carried vaccine serotypes decreased significantly across lineages; there was an increase of serotypes 7C, 15B/C, 23A, and 28F; antibiotic resistance rates remained stable; and certain accessory genes changed in frequency.

Pneumococcal conjugate vaccines (PCVs) have demonstrated high effectiveness on noninvasive [1] and invasive pneumococcal disease (IPD) [2]. Approximately 70% reduction of IPD was observed in the United States after 7-valent PCV (PCV7) introduction with >90% reduction of vaccine serotypes (VTs) [2]. PCV7 was not widely introduced in sub-Saharan Africa (SSA) due to predicted low VT coverage against common serotypes including 1 and 5 [3]. South Africa was among the few countries in SSA to implement PCV7, and a >85% reduction in IPD, which included human immunodeficiency virus (HIV)–infected individuals, was reported [4]. Subsequent higher-valent PCVs (10-valent PCV and 13-valent PCV [PCV13]) have shown high effectiveness in SSA in on VT carriage (>65%) [5] and IPD (>80%) in SSA [6, 7], consistent with reports in high-income countries [8].

While reduction of VT carriage has been documented post-PCV [9], the effect of PCV on overall carriage rates and density is not substantial [10, 11] because of serotype replacement post-PCV (increase of NVTs) [12, 13]. Such replacement is prompted by rewiring of strain competition dynamics when VTs become uncommon. The replacement NVTs can be novel (imported or capsule-switched) or extant due to clonal expansion of previously suppressed NVT serotypes. While replacement NVTs are less invasive than VTs, this is not universally true, and some strains retain propensity for IPD [14]. Therefore, post-PCV surveillance is crucial to monitor serotype frequency and interactions to inform optimal future PCV formulations. Pneumococcal surveillance typically focuses on IPD, but monitoring of carriage is also crucial as that is the niche where evolution occurs, therefore influencing population-level dynamics, unlike IPD, which is an evolutionary dead end.

PCV13 was introduced in Malawi on 12 November 2011 as an accelerated 3 + 0 schedule at 6, 10, and 14 weeks with a limited catch-up for infants in the first year from introduction. For the catch-up campaign, infants aged <12 months at the date of vaccine introduction were eligible for the following 12 months to receive 3 doses of PCV13, but coverage for the 3 doses of PCV13 was lower in catch-up infants (approximately 50%–70%) than in birth-eligible children (approximately 90%–95%) [15]. Recent carriage studies have reported a reduction of VTs but no overall change in carriage rates [16]. In this study, we undertook whole-genome sequencing (WGS) of pneumococcal isolates from northern Malawi to investigate early changes in population restructuring, within-lineage serotype composition, serotype diversity, antibiotic resistance (ABR), and accessory genome dynamics pre- and post-PCV. The WGS was part of the Global Pneumococcal Sequencing (GPS) project (www.pneumogen.net), which has sequenced approximately 23 000 isolates globally to study pneumococcal evolution patterns post-PCV to inform future vaccine design.

## MATERIALS AND METHODS

### Study Population and Isolate Selection

Household surveys of healthy carriers were conducted in the Karonga district of northern Malawi pre-PCV between 2009 and 2011 and post-PCV in 2014, and there were no significant changes of the overall pneumococcal carriage rates [16]. We randomly selected a subset of the samples (n = 660) pre-PCV in 2009–2010 (n = 482) and post-PCV in 2014 (n = 178) for a WGS survey (Supplementary Data 1). The mean age for samples collected pre-PCV was 6.24 (95% confidence interval [CI], 5.30–7.17) and 4.84 (95% CI, 3.79–5.89) post-PCV. The age ranged from 3 days to 54 years (pre-PCV) and 1 month to 30 years (post-PCV). In terms of age group, 506 samples collected pre-PCV were from children <5 years of age (n = 376; mean, 1.70 [95% CI, 1.53–1.86 ] years of age) and those ≥5 years of age (n = 130; mean, 22.33 [95% CI, 19.91–24.75] years of age) whereas 154 samples were collected post-PCV in those aged <5 years (n = 106; mean, 1.35 [95% CI, 1.05–1.65]) and ≥5 years (n = 48; mean, 14.29 [95% CI, 12.08–16.50]).

The nasopharyngeal swabs were processed as previously described [16]. Informed written consent was obtained from adults and from parents, guardians, and caregivers of child participants. The study was approved by the National Health Sciences Research Committee in Malawi (numbers 490 and 1232), London School of Hygiene and Tropical Medicine (number 5345), University of Liverpool (number 670), and University of Malawi College of Medicine Research Ethics Committee (number P.O8/14/1614).

### Genomic DNA Sequencing and Analysis

Procedures for genomic DNA extraction and sequencing were described previously [17]. The sequence reads were assembled using an automated assembly pipeline [18]. The serotypes and sequence types (STs) were identified using seroBA [19] and multilocus sequence typing [20], whereas ABR genes were detected using nucleotide-BLAST version 2.2.30 (*E* value < 0.001, sequence coverage and identity >80%) [21]. Penicillin minimum inhibitory concentrations (MICs) were genotypically predicted using the Centers for Disease Control and Prevention pipeline [22] and the MICs were interpreted using the British Society for Antimicrobial Chemotherapy breakpoints [23]. The sequence reads were deposited in the European Nucleotide Archive (accession numbers are shown in Supplementary Data 1).

Genomic clusters (GCs) or lineages were inferred using Bayesian Analysis of Population Structure (BAPS) version 6.0 [24]. A 1 050 021 bp core-gene alignment with 88 961 single-nucleotide polymorphism (SNP) positions was generated using Roary [25]. Phylogenetic trees for all isolates were constructed using the core-gene alignment using FastTree-SSE3 version 2.1.3 [26] whereas lineage-based trees used consensus alignments from SMALT (https://sourceforge.net/projects/smalt/) for Gubbins version 1.4.10 [27] and RAxML version 7.0.4 [28]. The SNPs were reconstructed on the trees using parsimony. The trees were visualized using iToL version 2.1 [29] and MicroReact [30].

### Statistical Analysis

Serotype and ABR gene frequencies were compared using Fisher exact test. The Simpson diversity index (D) for serotype and ST composition was estimated using a web-based analysis tool (www.comparingpartitions.info). The differences in penicillin MICs were assessed using Student *t* test. The binary presence-absence of accessory genes pre- and post-PCV was assessed using logistic regression, which controlled for vaccine status and age group of the isolates with Bonferroni correction for multiple testing. We used R version 3.1.2 for statistical analyses (R Core Team, 2013, www.r-project.org).

## RESULTS

### Defining the Population Structure

**Figure 1.**
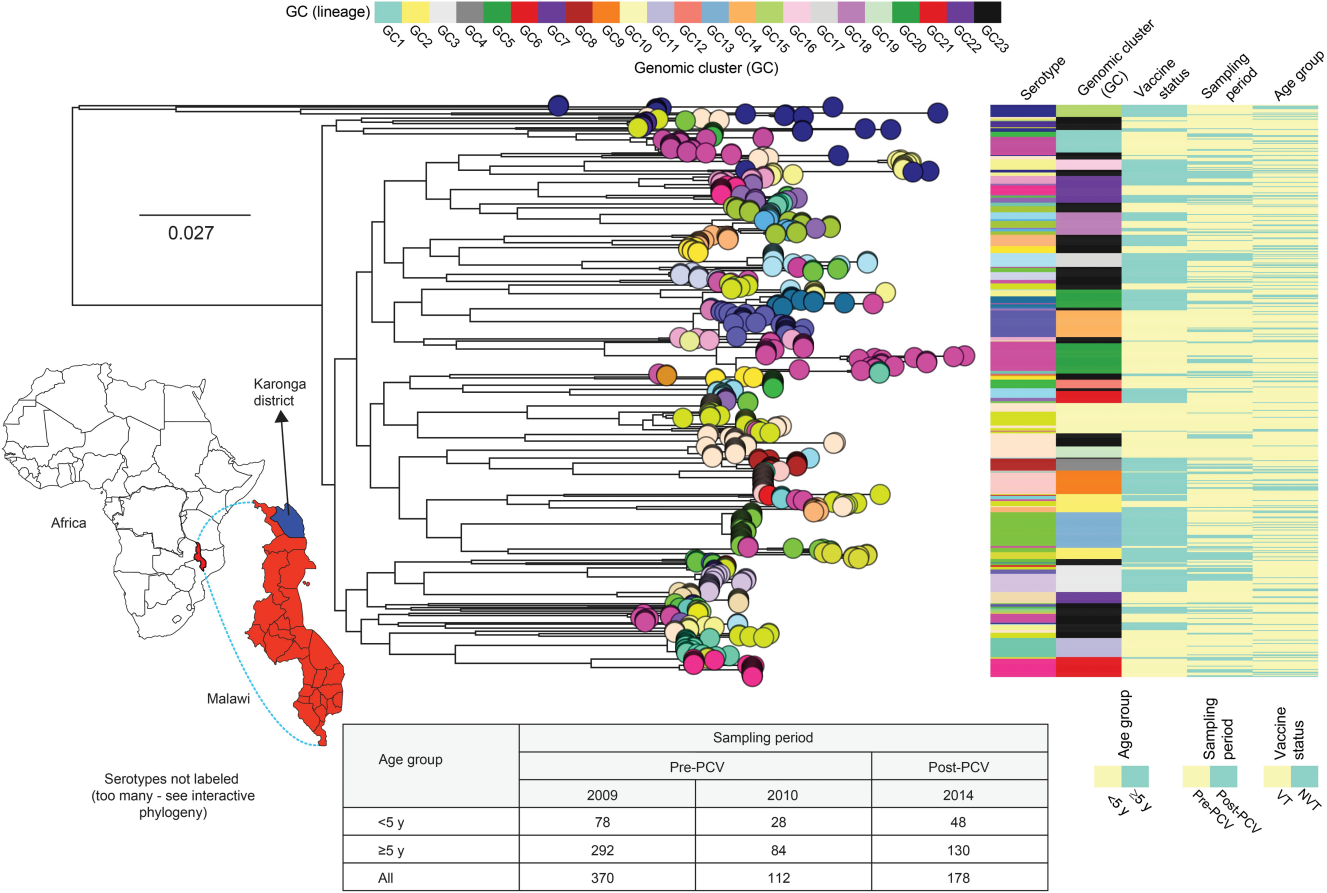
Sampling location, genetic similarity, and distribution of carried pneumococcal isolates. The map of Africa shows the location of Malawi and Karonga district from which the isolates were sampled. The number of isolates (n = 660) used in the genomic analysis are shown in the table below the phylogenetic tree. The core genome maximum likelihood phylogenetic tree of the 660 carriage isolates rooted at the branch of “classical” nontypeables. The tips (circles) of the tree are colored by serotype, and colored panels to the right correspond to serotype, genomic clusters or lineage, vaccine status, sampling period, and age group. The tree with metadata and corresponding international definitions of the pneumococcal lineages is available interactively online at https://microreact.org/project/xH7-VcoWj/8a339d57. Abbreviations: GC, genomic cluster; NVT, nonvaccine serotype; PCV, pneumococcal conjugate vaccine; VT, vaccine serotype.

Whole-genome sequences for the 660 carried isolates revealed 45 serotypes and 169 STs (Supplementary Data 1). The majority of the serotypes were associated with a single phylogenetic tree branch with few serotypes dispersed across multiple unrelated branches due to capsule switching (acquisition of the capsule/serotype in unrelated strains). To account for genetic background of the isolates, we defined 23 lineages or GCs using unsupervised nucleotide sequence clustering, and these lineages allowed for subsequent comparison of serotype changes in context of their lineage membership (Figure 1A). Phylogenetically, all of the lineages (GC1–GC22) except for GC23 were monophyletic, with their members emerging from a single recent common ancestor. The defined lineages varied in composition of serotypes and sequence diversity reflecting differences in either evolution rates (higher rate imply higher diversity) or age (older lineages accrue more diversity). An interactive phylogenetic tree of the isolates is available in MicroReact (https://microreact.org/project/xH7-VcoWj/8a339d57).

### Frequency of Vaccine Serotypes and Their Associated Lineages

**Figure 2.**
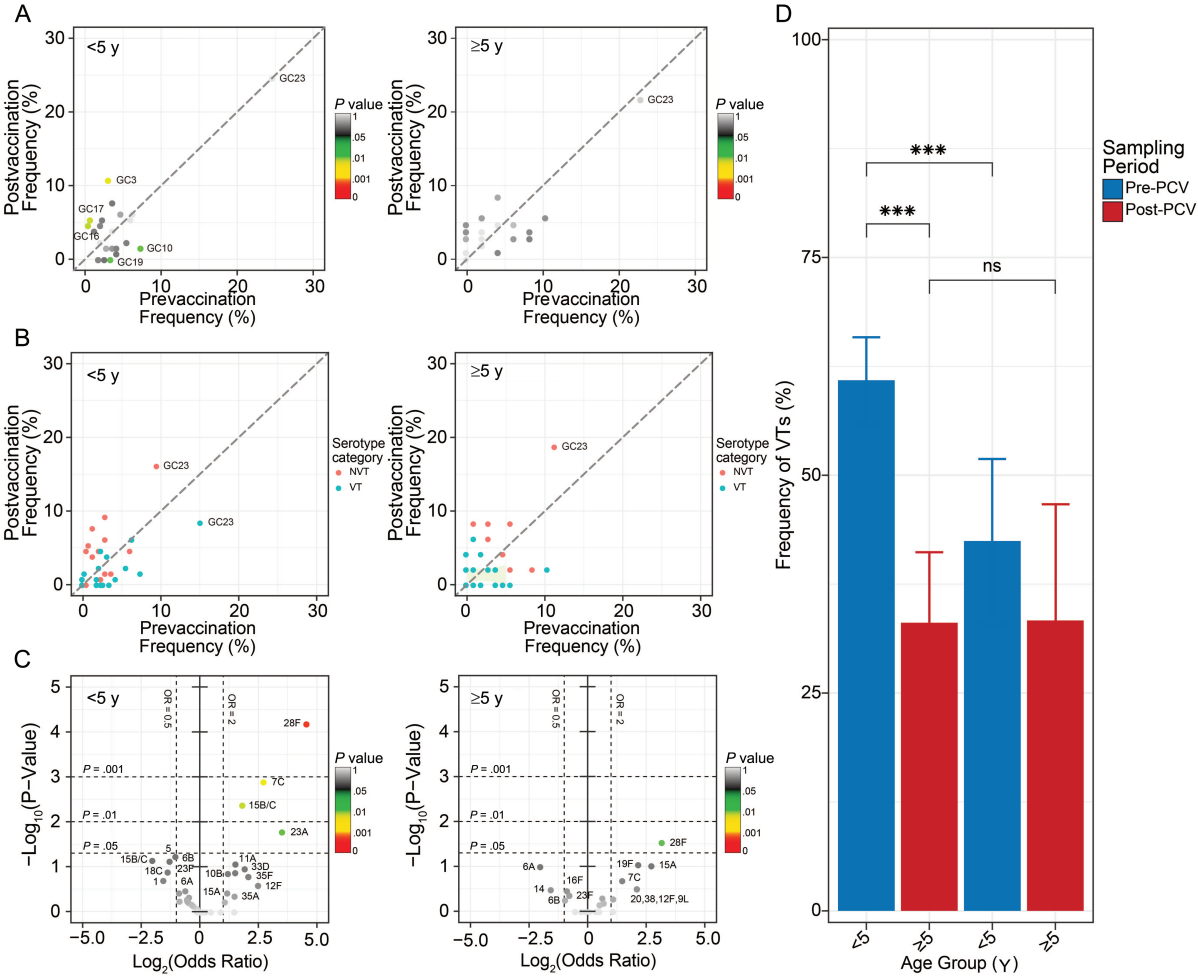
Frequency of lineages and serotypes in carriage. *A* and *B*, Frequency of lineages (*A*) and frequency of vaccine serotypes (*B*) before and after pneumococcal conjugate vaccine (PCV) in individuals <5 and ≥5 years of age. *C*, Volcano plots showing odds ratios (ORs) of individual serotypes pre- and post-PCV in those <5 and ≥5 years of age. The x-axis shows magnitude (log_2_ [OR]) and y-axis shows statistical significance (–log_10_*P* value). *D*, Frequency of vaccine serotypes in those <5 and ≥5 years of age. Statistically significant changes are marked as ***P < .001. The comparative estimates of prevalence for serotypes, lineages, and ORs are provided in Supplementary Tables 1–5. Abbreviations: GC, genomic cluster; ns, not significant; NVT, nonvaccine serotype; PCV, pneumococcal conjugate vaccine; VT, vaccine serotype.

The frequency of lineages GC3 (*P* = .002), GC16 (*P* = 0.004), and GC17 (*P* = .004) in those <5 years of age increased post-PCV whereas lineages GC10 (*P* = .016) and GC19 (*P* = .026) showed a decrease in the same age group (Figure 2A; Supplementary Table 1). These were predominantly VT-associated lineages; therefore, their decreased frequency reflects reduction of VTs in isolates sampled from those <5 years of age, who composed the majority of vaccinated individuals, but this reduction was not seen in the unvaccinated population ≥5 years of age (Figure 2B; Supplementary Table 2). However, despite the observed decrease in frequency of VTs in lineages, the odds ratio (OR) for VT serotypes in each lineage in children <5 years of age relative to individuals aged ≥5 years remained unchanged post-PCV except for lineages GC4 and GC16, in which the OR for VTs increased, and lineages GC19 and GC21, where the OR decreased (Supplementary Table 3). By individual serotypes, frequency of multiple NVTs increased post-PCV, namely serotypes 7C (*P* = .001), 15B/C (*P* = .004), 23A (*P* = .017), and 28F (*P* = .0001) in those <5 years of age and 28F (*P* = .029) in persons ≥5 years of age (Figure 2C; Supplementary Table 4). The overall frequency of VTs regardless of lineages decreased post-PCV in children aged <5 years (60.08% to 33.08%; *P* = 4.80 × 10^–8^) but not in those aged ≥5 years (42.45% to 33.33%; *P* = .3739) (Figure 2D; Supplementary Table 5). However, the frequency of VTs was higher in those <5 years of age compared with those ≥5 years of age (*P* = 8.44 × 10^–4^) pre-PCV, but no differences were observed post-PCV (Figure 2D; Supplementary Tables 4 and 5).

### Emergence and Clonal Expansion of NVT Isolates

**Figure 3.**
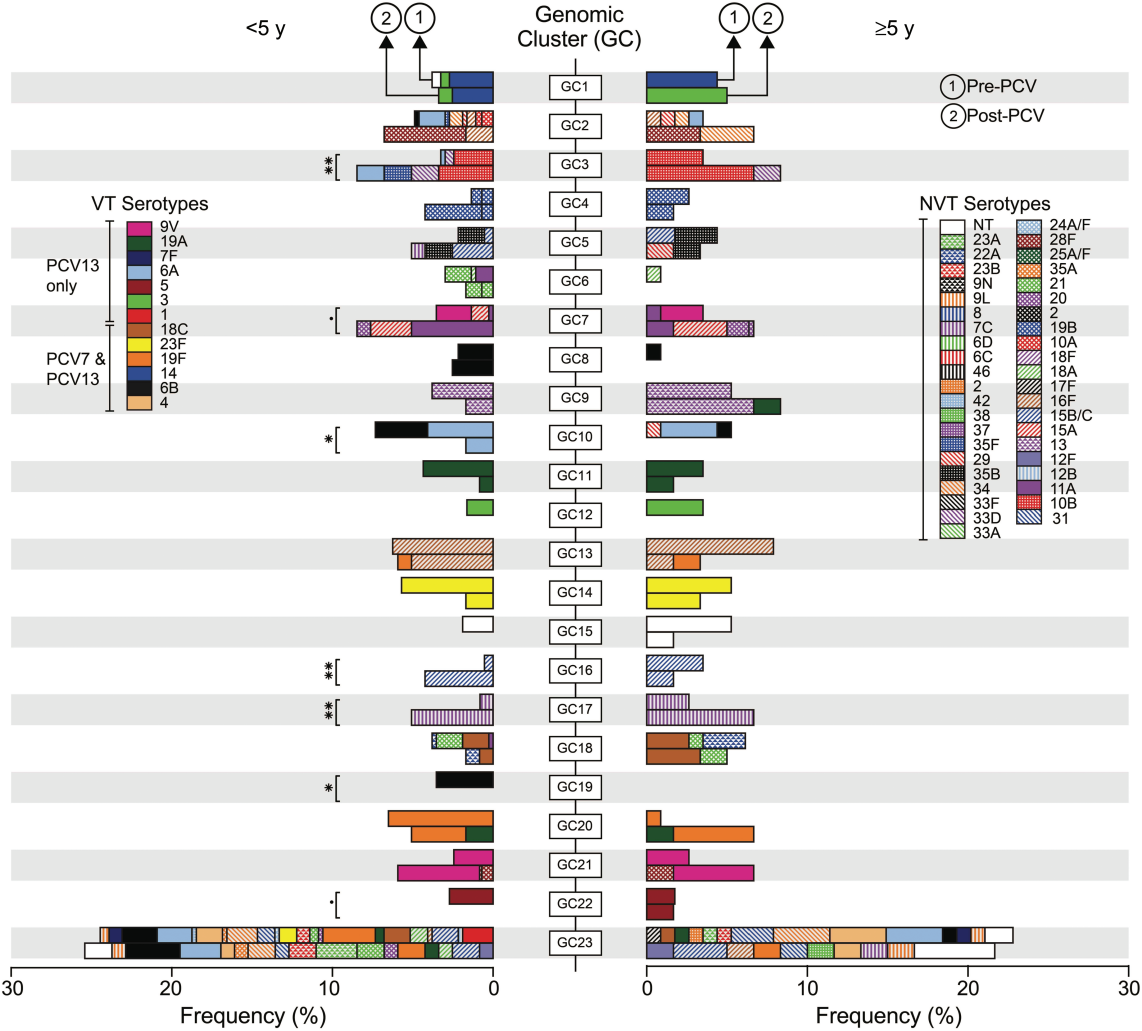
Dynamics of pneumococcal lineages and serotypes. The leftward facing stacked bar graph shows frequency of lineages in individuals <5 years while the rightward facing bar graph shows frequency of lineages and their constituent serotypes in those aged ≥5 years before and after pneumococcal conjugate vaccine (PCV) introduction. The bar graphs are aligned by genomic clusters (GCs) for easy comparisons of frequency of serotypes pre- and post-PCV between the 2 age groups. The serotypes are distinguished by different colors in the bar graphs as described in the key. The GC23 is the “bin” cluster because it consists of isolates not placed in monophyletic clusters GC1–GC22. The lineages whose frequency changed significantly post-PCV are marked as *P < .05 and **P < .01, and those with borderline significance (*P* < .095) are marked with (.). The Fisher exact test was used to determine *P* values. Abbreviations: NT, nontypeable; PCV7, 7-valent pneumococcal conjugate vaccine; PCV13, 13-valent pneumococcal conjugate vaccine; VT, vaccine serotype.

**Figure 4.**
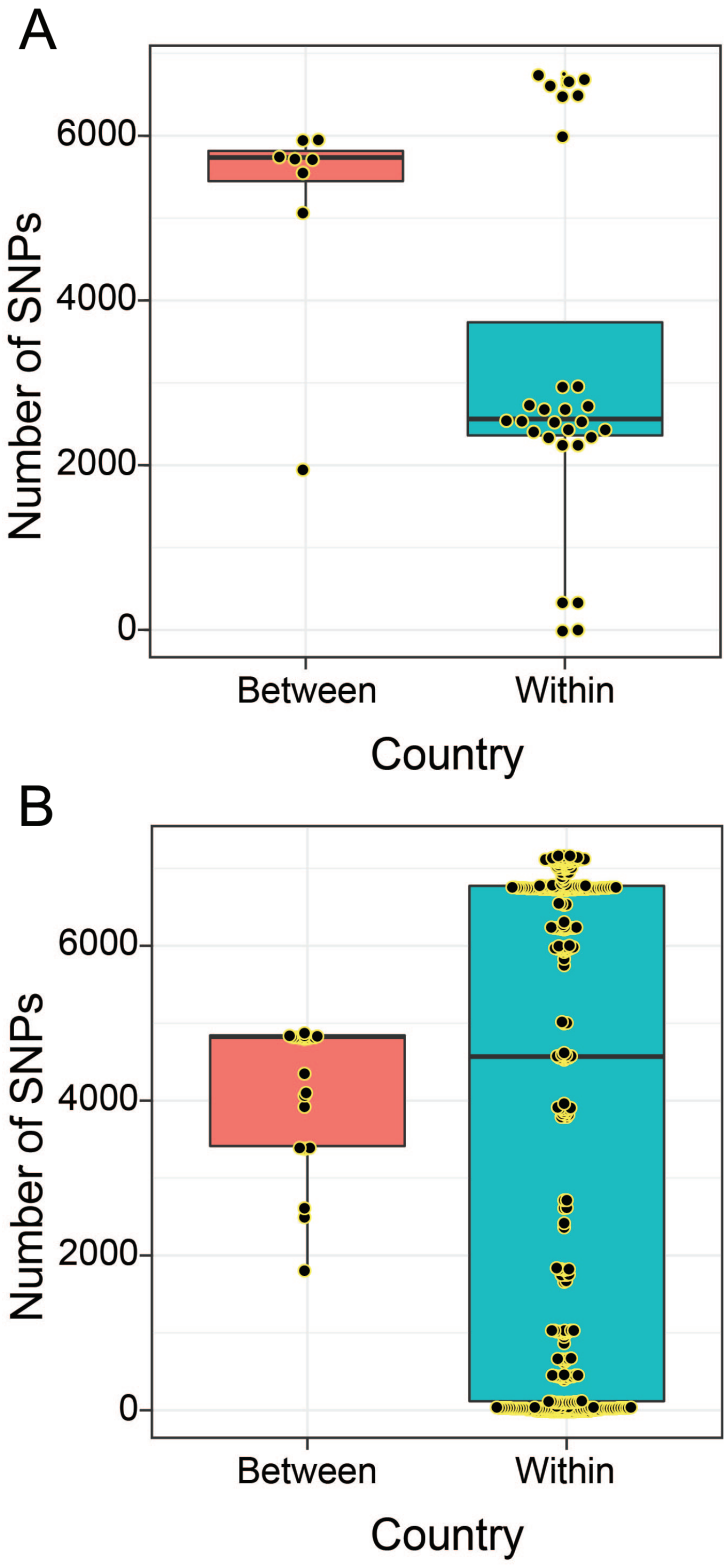
Genetic diversity of a recently emerged serotype (28F). Boxplots showing within (Malawi) and between country (Malawi and South Africa) genetic diversity of serotype 28F isolates showing in GC2 (*A*) and GC21 (*B*). Lineage GC21 also includes serotype 9V isolates, some of which underwent a capsule switch to acquire a serotype 28F capsule. Abbreviation: SNP, single-nucleotide polymorphism.

Within-lineage dynamics revealed how serotype frequencies were changing post-PCV. For example, clonal expansion of serotypes 11A (GC7) and 15B/C (GC16) occurred post-PCV (Figure 3). By comparing pre- and post-PCV frequencies of serotypes in the lineages it was clear that the majority of the serotypes were extant pre-PCV whereas a few were first detected post-PCV in some lineages, for example, serotype 19F (GC13) and 19A (GC20), which were capsule-switch events. Comparison of overall serotype frequencies revealed that serotype 28F (GC2 and GC21) was the only serotype not detected pre-PCV, which posed questions regarding whether it emerged by recent importation or post-PCV unmasking after prior circulation pre-PCV at undetectable level. Genomic analysis showed that GC21 contained serotype 9V and that the 28F among 9V strains in GC21 emerged post-PCV by a 9V→28F vaccine capsule switch; the capsule-switched isolates were distinguished by approximately 20 SNPs within themselves and with closest 9V isolates. However, accrued genetic diversity of 28F isolates in GC2 was higher than expected to occur under pneumococcal mutation rate if the isolates emerged post-PCV (maximum 6757 SNPs) (Figure 4). Because recently imported isolates typically undergo a loss in genetic diversity (bottleneck), our findings were not consistent with this, therefore implying that serotype 28F isolates existed at undetectable levels pre-PCV and were unmasked due to clearance of VTs post-PCV, which subsequently led to the serotype 9V→28F vaccine capsule-switch in GC21. There was further evidence against importation from other countries because serotype 28F with similar genetic profiles were uncommon globally (2/12000 in GPS collection); these differed from our isolates by >2000 SNPs and therefore could not be the source. Additional capsule-switched isolates detected post-PCV only included 11A→20 (GC7), 13→19A (GC9), 16F→19F (GC13), (GC21), and 7C→NT (GC23), but none of these were vaccine-escape events.

### Serotype Diversity as an Indicator for PCV Effectiveness

**Figure 5.**
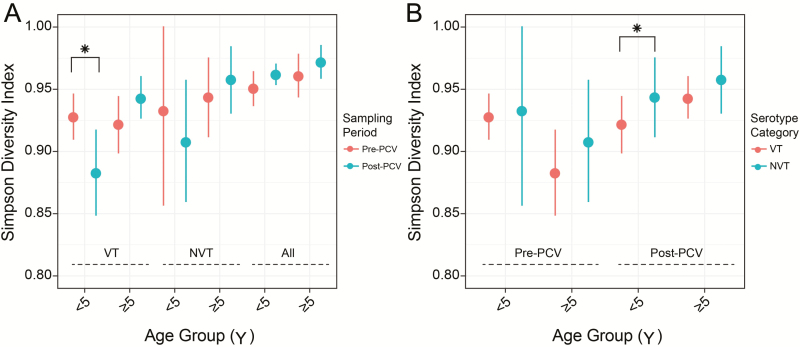
Serotype composition and diversity in context of pneumococcal conjugate vaccine (PCV). *A*, Simpson diversity index for composition of serotypes between pre- and post-PCV datasets among vaccine serotypes (VT), nonvaccine serotypes (NVT), and all isolates. *B*, Simpson diversity index for composition of serotypes between VT and NVT isolates sampled pre- and post-PCV. Statistically significant changes are marked as *P < .05. The estimates and *P* values for frequency of VTs and Simpson diversity are provided in Supplementary Tables 6 and 7.

The pre-PCV equilibrium diversity of serotypes is altered by PCV; therefore, changes in this diversity in VTs and NVTs quantified using the Simpson diversity index (D) can signal population-level PCV effectiveness. In this study, Simpson D for serotypes decreased post-PCV in VTs in children <5 years of age (*P* = .022) but not in those ≥5 years of age (Figure 4A; Supplementary Table 6). However, Simpson D appeared to decrease and increase in NVTs in those <5 and ≥5 years of age, respectively, although this was not statistically significant (Figure 5). The Simpson D for serotypes was similar between VTs and NVTs pre-PCV, but it was higher in NVTs than VTs post-PCV (*P* = .004) (Figure 5B). The Simpson D was higher for STs than serotypes both pre-PCV (*P* = .011) and post-PCV (*P* = 0) but remained unchanged post-PCV; therefore, ST diversity may not be informative of PCV effect (Supplementary Tables 6 and 7).

### Frequency of Antibiotic Resistance and Other Accessory Genes

**Figure 6.**
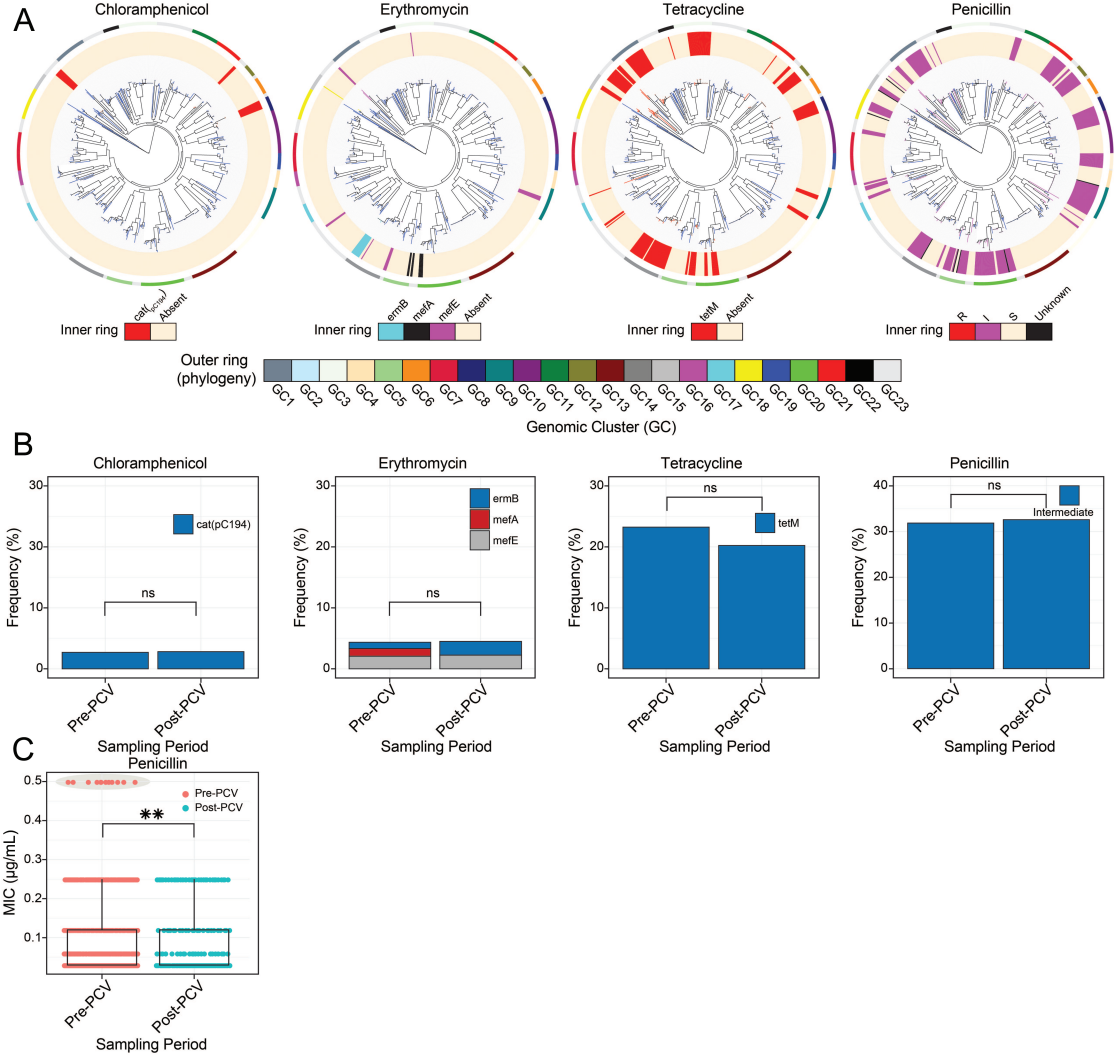
Distribution of antibiotic resistance (ABR) genes and mobile genetic elements before and after pneumococcal conjugate vaccine (PCV) introduction. *A*, Distribution of the chloramphenicol resistance gene (*cat*_pC194_), erythromycin resistance genes (*mefA, mefE*, and *ermB*), tetracycline resistance gene (*tetM*), and penicillin resistance genes across the phylogenetic tree of the carried pneumococcal isolates. Presence and absence of genes is indicated by colored branches and innermost ring surrounding the phylogeny as shown in the key at the bottom of each tree. *B*, Frequency of genotypic ABR rates for chloramphenicol, erythromycin, tetracycline, and intermediate-penicillin-resistance pre- and post-PCV. *C*, Distribution of penicillin minimum inhibitory concentration pre- and post-PCV. The subsets with statistically significant changes are marked as **P < .01. The estimates for frequency of the ABR genes are provided in Supplementary Table 8. Abbreviations: GC, genomic cluster; MIC, minimum inhibitory concentration; ns, not significant; PCV, pneumococcal conjugate vaccine.

An important subset of pneumococcal accessory genes encodes for ABR-conferring proteins; therefore, we assessed their distribution pre- and post-PCV. The genes confer resistance against tetracycline, chloramphenicol, and erythromycin. No significant changes in frequency of ABR genes (rates) occurred post-PCV (Figure 6A and 6B; Supplementary Table 8). We also assessed MIC changes in penicillin resistance genotypically, and resistance rates were similarly unchanged, although the MICs decreased post-PCV (*P* = .0098) due to clearance of intermediate-resistant VT isolates in GC12 (Figure 6C).

**Figure 7.**
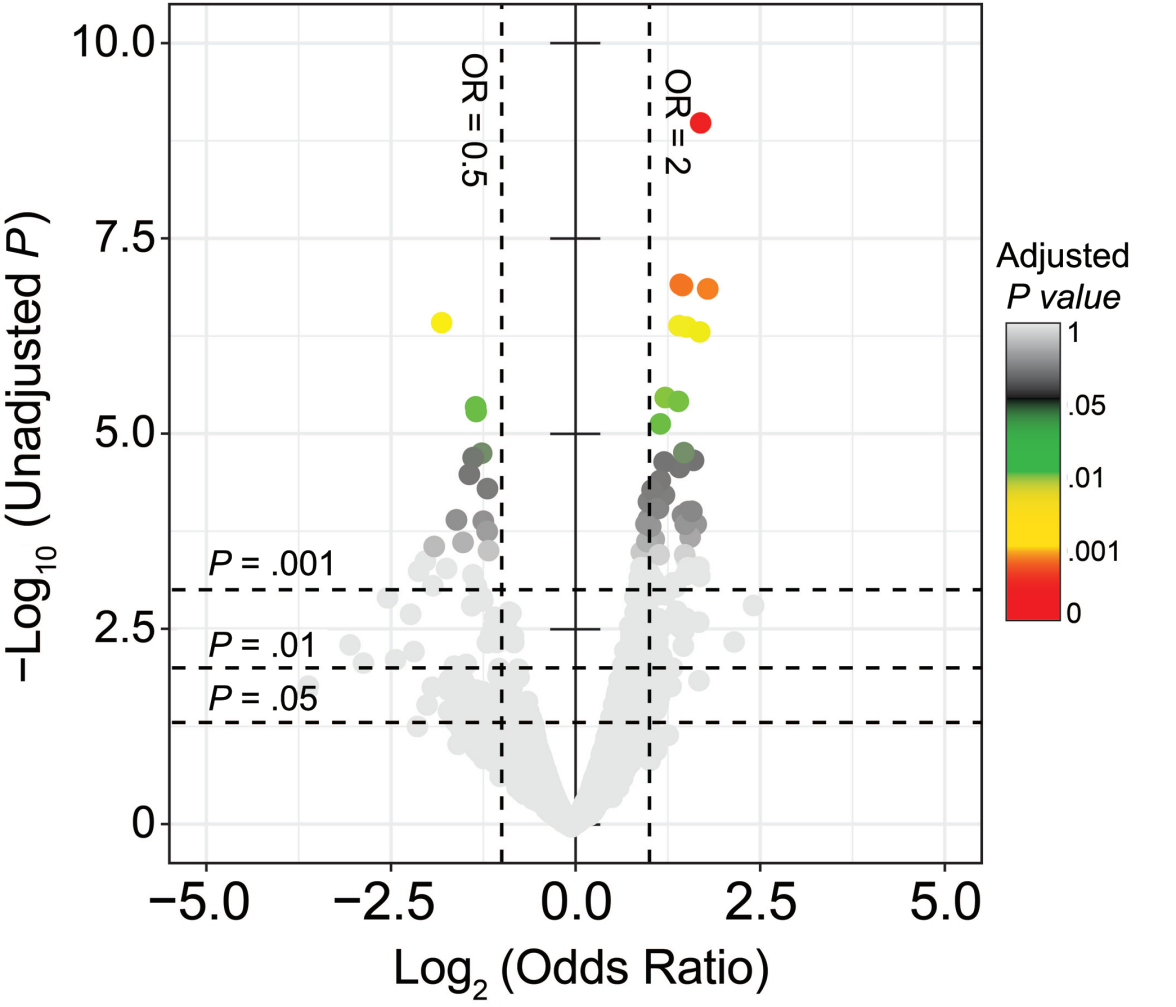
Pneumococcal accessory genome dynamics. The distribution of 2591 intermediate-frequency-accessory genes in the entire pneumococcal population. The volcano plot shows magnitude (log_2_ OR) on the x-axis and statistical significance (–log_10_*P* value) and odds ratio (OR) for presence of accessory genes post–pneumococcal conjugate vaccine (PCV) relative to pre-PCV after controlling for vaccine status and age group of the isolates. The points were colored by adjusted *P* values after correcting for multiple testing using Bonferroni method. The estimates for the OR and *P* values are provided in Supplementary Table 9.

To assess whether other accessory genes had changed in frequency post-PCV unlike ABR genes, logistic regression model was fitted to binary gene presence-absence data with sampling period of the isolates as independent variable adjusted for PCV status and age group. Significant changes in frequency were detected in 42 accessory genes post-PCV after Bonferroni correction (Figure 7; Supplementary Table 9). There was an increasing trend in half of the genes with lowest *P* values associated with glycosyl transferase (OR, 3.34; *P* = 9.71 × 10^–6^), bacteriolysin (OR, 3.34; *P* = 3.46 × 10^–5^), and restriction modification system (OR, 3.34; *P* = 3.46 × 10^–5^). Conversely, bacteriocin gene *blpQ* (OR, 0.19; *P* = 5.20 × 10^–6^) showed the most significant decrease. The capsule biosynthesis gene *wzx* also decreased post-PCV (OR, 0.43; *P* = .042). By functional classification, the majority of the detected genes were associated with mobile DNA (11/42) and bacteriocins (3/42).

## DISCUSSION

Pneumococcal evolution occurs during carriage; therefore, monitoring carriage population is crucial to understand ongoing strain dynamics and adaptations post-PCV [4, 5]. Our genomic study demonstrates the utility of WGS in monitoring changes in pneumococcal carriage in low-income settings after PCV introduction. We provide evidence of early pneumococcal population restructuring using WGS observed beyond serotype but also at the lineage and accessory genome level in both children and adults 2 years after PCV introduction. The most significant finding is that decrease of VTs occurred consistently across their associated lineages, reflecting no interference of genetic background on PCV effectiveness; most importantly, the collective decrease of VTs was more pronounced in children <5 years of age, which implies high direct PCV effects consistent with data from clinical trials [16]. However, our findings show modest decrease in frequency of VTs and VT-associated lineages in the population ≥5 years of age, which implies either limited or delayed indirect PCV effects in contrast to other settings where higher indirect effects have been demonstrated in older populations [9, 31]. It is currently unclear whether limited indirect PCV effects in our setting are related to PCV scheduling or mitigating factors for herd effects such as HIV-infected adults considered as potential reservoirs for pneumococcal diversity post-PCV [32].

PCV implementation changes both the frequency of individual serotypes and their composition in the population; therefore, quantifying the degree of disorder of serotypes with Simpson D can indirectly inform population-level PCV effects [33, 34]. Our findings showed that serotype diversity in VTs decreased significantly post-PCV but only in the under-5 population, which implies substantial loss of diversity in VTs in vaccinated individuals <5 years of age, but not older unvaccinated individuals, consistent with the serotype frequency–based observation of high direct PCV effect but limited indirect effects. We expected an increase in serotype diversity in NVTs due to serotype replacement; on the contrary, this did not occur in either age group, which implies that although the overall carriage rate was rapidly restored by replacement NVTs, these resulted in marginal accrual of additional diversity in NVTs post-PCV. This may be a consequence of incomplete serotype replacement process because the isolates were sampled only 2 years after PCV introduction. However, increase in frequency of individual NVTs occurred for serotypes 7C, 23A, and 15B/C in those aged <5 years and serotype 28F in both age groups, and these have been reported elsewhere except for serotype 28F [35, 36]. Unlike other serotypes, 28F was detected only post-PCV in lineages GC2 and GC21, which initially prompted speculation that it was imported from another country. However, within-lineage genetic diversity of 28F isolates and genetic dissimilarity from similar isolates in the GPS collection ruled out importation because the genetic diversity was much higher than can be expected for a newly introduced clone, which suggested that it had been circulating pre-PCV but at undetectable levels. Interestingly, the serotype 28F in GC21 emerged post-PCV via a vaccine-escape 9V→28F capsule switch between unmasked 28F in GC2 and 9V isolates in GC21, but there were no other vaccine-escape capsule switches detected for other serotypes. Therefore, the rarity of vaccine-escape capsule switches demonstrates negligible effect of capsule-switching process on serotype replacement, unlike clonal expansion of previously masked capsule-intact NVT isolates.

The pneumococcal accessory gene pool consists of a diverse repertoire of genes, which include ABR, mobile genetic element (MGE), and competition-associated genes. We noted stability in frequency of ABR genes post-PCV but considering that the levels were already low, unlike in IPD isolates in Malawi, this did not raise concerns [37–39]. Interestingly, PCV reduced frequency of intermediate-penicillin-resistant isolates associated with VT lineages, which showcases how PCV can be strategically harnessed as a preventive strategy to thwart emergence of high resistance by targeting low-level resistance lineages with highest likelihood to express full resistance in the future [40]. Other non-ABR-associated accessory genes showed changes in frequency post-PCV after controlling for age group and vaccine status of the isolates; these predominantly included highly mobile and rapidly shared MGE-associated genes and bacteriocin immunity proteins that mediate isolate competition, therefore suggesting potential benefits in emerging NVTs as the pneumococcal population reestablishes equilibrium serotype dynamics.

Our findings demonstrate early changes in the pneumococcal carriage population in a low-income setting after PCV introduction. Our findings provide the first large-scale post-PCV genomic survey of carried pneumococcal isolates in an African setting where, despite high disease burden, limited WGS studies are conducted. Therefore, this study provides useful baseline data for comparative analyses of population-level effects of PCV between different settings, both low and high income. The play of chance and possibly small sample sizes for subset analyses could have impacted some of our findings, so cautious interpretation is recommended. Continued diligent surveillance and WGS remain crucial for monitoring long-term residual effects of VTs, serotype replacement, and genotypic changes post-PCV after equilibrium serotype dynamics are reestablished. Additionally, our study complements vaccine efficacy data from clinical trials and therefore improves our understanding of population-level effects of PCV in Malawi, SSA, and globally, which will help to inform optimal combination of serotypes for future PCVs to maximize their beneficial effects, especially in vulnerable tropical populations.

## Supplementary Data

Supplementary materials are available at *Clinical Infectious Diseases* online. Consisting of data provided by the authors to benefit the reader, the posted materials are not copyedited and are the sole responsibility of the authors, so questions or comments should be addressed to the corresponding author.

ciz404_suppl_Supplementary_Data

ciz404_suppl_Supplementary_Material

## Notes

***Author contributions.*** C. C., N. F., D. B. E., and S. D. B. conceived and designed the study. N. F., D. B. E., and S. D. B. supervised the study. E. H., T. T., and N. F. conducted the field studies. M. A., A. W. K., J. E. C., and C. P. performed molecular and microbiology experiments. S. D. B. supervised whole-genome sequencing and genomic analysis. C. C. and R. A. G. checked quality of the sequence assemblies. C. C. performed genomic and statistical analyses. C. C. and S. D. B. wrote the initial draft of the manuscript. P. M., L. M., R. F. B., A. K., W. P. H., and R. S. H. contributed to data interpretation. All authors contributed to writing and review of the manuscript.

***Acknowledgments.*** The authors acknowledge work by clinical and laboratory staff at Malawi Epidemiology and Intervention Research Unit and Malawi-Liverpool-Wellcome Trust Clinical Research Programme (MLW) and the sequencing and informatics teams at Wellcome Sanger Institute.

***Financial support.*** This work was supported by the Bill & Melinda Gates Foundation’s funding for the Global Pneumococcal Sequencing (GPS) project (www.pneumogen.net; grant number OPP1034556 to S. D. B., L. M., and R. F. B.) and by Wellcome UK (core grant number 084679/Z/08/Z to the MLW). R. S. H. and N. F. are supported by the UK Medical Research Council (MRC) and the UK Department for International Development (DFID) grant under the MRC/DFID Concordat agreement and is also part of the EDCTP2 program supported by the European Union (MR/N023129/1). C. C. was funded by a PhD studentship from the Commonwealth Scholarship Commission in the United Kingdom.

***Potential conflicts of interest.*** N. B. Z. has received investigator-initiated project grants from GlaxoSmithKline Biologicals and from Takeda Pharmaceuticals. R. A. G. has received a PhD studentship from Pfizer in 2009–2013. W. P. H. has received personal fees from Antigen Discovery. All other authors report no potential conflicts of interest. All authors have submitted the ICMJE Form for Disclosure of Potential Conflicts of Interest. Conflicts that the editors consider relevant to the content of the manuscript have been disclosed.
