# Supplementary material for: Early Signals of Vaccine-driven Perturbation Seen in Pneumococcal Carriage Population Genomic Data
Source: Clin Infect Dis. 2019 May 16;70(7):1294–303. doi: 10.1093/cid/ciz404 (PMC7768739; doi:10.1093/cid/ciz404)
Supplement: ciz404_suppl_Supplementary_Material [file ciz404_suppl_supplementary_material.doc]

**Supplementary Table 1. Frequency of pneumococcal genomic clusters in carried pneumococcal population pre- and post-vaccination.**

| **Genomic cluster (GC)** | **Frequency in**  **under-five (%)** | | **Odds ratio** | **P-Value*** | **Frequency in**  **over-five (%)** | | **Odds ratio** | **P-Value*** |
| --- | --- | --- | --- | --- | --- | --- | --- | --- |
|  | **Pre-vaccination** | **Post-vaccination** |  |  | **Pre-vaccination** | **Post-vaccination** |  |  |
| GC1 | 3.72 (14/376) | 3.85 (5/130) | 1.15 | 1 | 4.72 (5/106) | 4.17 (2/48) | 1.09 | 1 |
| GC2 | 0.05 (18/376) | 6.15 (8/130) | 1.38 | 0.4996 | 3.77 (4/106) | 8.33 (4/48) | 2.29 | 0.2568 |
| GC3 | 0.03 (12/376) | 10.77 (14/130) | 3.60 | 0.0019 | 3.77 (4/106) | 2.08 (1/48) | 0.86 | 1 |
| GC4 | 0.01 (5/376) | 3.85 (5/130) | 2.95 | 0.1341 | 2.83 (3/106) | 2.08 (1/48) | 1.08 | 1 |
| GC5 | 0.02 (8/376) | 4.62 (6/130) | 2.30 | 0.2094 | 4.72 (5/106) | 4.17 (2/48) | 1.09 | 1 |
| GC6 | 0.03 (11/376) | 1.54 (2/130) | 0.71 | 0.5303 | 0.94 (1/106) | 0 (0/48) | 1.08 | 1 |
| GC7 | 0.04 (14/376) | 7.69 (10/130) | 2.20 | 0.0908 | 2.83 (3/106) | 8.33 (4/48) | 2.89 | 0.2051 |
| GC8 | 0.02 (8/376) | 2.31 (3/130) | 1.28 | 1 | 0.94 (1/106) | 0 (0/48) | 1.08 | 1 |
| GC9 | 0.04 (14/376) | 1.54 (2/130) | 0.56 | 0.3812 | 5.66 (6/106) | 10.42 (5/48) | 1.97 | 0.3197 |
| GC10 | 0.07 (28/376) | 1.54 (2/130) | 0.28 | 0.0157 | 4.72 (5/106) | 0 (0/48) | 0.35 | 0.3253 |
| GC11 | 0.04 (16/376) | 0.77 (1/130) | 0.33 | 0.0856 | 3.77 (4/106) | 2.08 (1/48) | 0.86 | 1 |
| GC12 | 0.02 (7/376) | 0 (0/130) | 0.35 | 0.1990 | 2.83 (3/106) | 0 (0/48) | 0.53 | 0.5525 |
| GC13 | 0.06 (23/376) | 5.38 (7/130) | 0.95 | 1 | 8.49 (9/106) | 4.17 (2/48) | 0.63 | 0.5041 |
| GC14 | 0.06 (21/376) | 2.31 (3/130) | 0.51 | 0.1555 | 5.66 (6/106) | 2.08 (1/48) | 0.60 | 0.4358 |
| GC15 | 0.02 (7/376) | 0 (0/130) | 0.35 | 0.1990 | 5.66 (6/106) | 2.08 (1/48) | 0.60 | 0.4358 |
| GC16 | 0.01 (2/376) | 4.62 (6/130) | 7.00 | 0.0046 | 3.77 (4/106) | 0 (0/48) | 0.42 | 0.3105 |
| GC17 | 0.01 (3/376) | 5.38 (7/130) | 6.03 | 0.0038 | 2.83 (3/106) | 6.25 (3/48) | 2.26 | 0.3764 |
| GC18 | 0.04 (16/376) | 1.54 (2/130) | 0.49 | 0.1795 | 4.72 (5/106) | 6.25 (3/48) | 1.48 | 0.7050 |
| GC19 | 0.03 (13/376) | 0 (0/130) | 0.20 | 0.0261 | 0 (0/106) | 0 (0/48) | 2.18 | 1 |
| GC20 | 0.06 (24/376) | 6.15 (8/130) | 1.03 | 1 | 0.94 (1/106) | 4.17 (2/48) | 3.38 | 0.2293 |
| GC21 | 0.02 (9/376) | 5.38 (7/130) | 2.37 | 0.1404 | 2.83 (3/106) | 8.33 (4/48) | 2.89 | 0.2051 |
| GC22 | 0.03 (10/376) | 0 (0/130) | 0.25 | 0.0712 | 1.89 (2/106) | 2.08 (1/48) | 1.46 | 1 |
| GC23 | 0.25 (93/376) | 24.62 (32/130) | 1.01 | 1 | 21.70 (23/106) | 22.92 (11/48) | 1.11 | 0.8375 |
| *P-values were calculated using the Fisher’s Exact Test. | | | | | | | | |

**Supplementary Table 2. Frequency of vaccine type (VT) and non-vaccine type (NVT) pneumococcal strains in genomic clusters (GC) among carried pneumococcal strains pre- and post-vaccination.**

| **Age group (years)** | **Genomic cluster (GC)** | **Vaccine type (VT %)** | | **P-Value*** |  | **Non-vaccine type (NVT %)** | | | **P-Value*** |
| --- | --- | --- | --- | --- | --- | --- | --- | --- | --- |
|  |  | **Pre-vaccination** | **Post-vaccination** |  |  | **Pre-vaccination** | **Post-vaccination** |  | |
| **Under-five (<5 years)** | 1 | 3.19 (12/376) | 3.85 (5/130) | 0.7782 |  | 0.53 (2/376) | 0 (0/130) | 1 | |
|  | 2 | 1.86 (7/376) | 0 (0/130) | 0.1990 |  | 2.93 (11/376) | 6.15 (8/130) | 0.1095 | |
|  | 3 | 0.27 (1/376) | 1.54 (2/130) | 0.1635 |  | 2.93 (11/376) | 9.23 (12/130) | 0.0058 | |
|  | 4 | 0 (0/376) | 0 (0/130) | 1 |  | 1.33 (5/376) | 3.85 (5/130) | 0.1341 | |
|  | 5 | 0 (0/376) | 0 (0/130) | 1 |  | 2.13 (8/376) | 4.62 (6/130) | 0.2094 | |
|  | 6 | 0 (0/376) | 0 (0/130) | 1 |  | 2.93 (11/376) | 1.54 (2/130) | 0.5303 | |
|  | 7 | 2.39 (9/376) | 0 (0/130) | 0.1203 |  | 1.33 (5/376) | 7.69 (10/130) | 0.0008 | |
|  | 8 | 2.13 (8/376) | 2.31 (3/130) | 1 |  | 0 (0/376) | 0 (0/130) | 1 | |
|  | 9 | 0 (0/376) | 0 (0/130) | 1 |  | 3.72 (14/376) | 1.54 (2/130) | 0.3812 | |
|  | 10 | 7.45 (28/376) | 1.54 (2/130) | 0.0157 |  | 0 (0/376) | 0 (0/130) | 1 | |
|  | 11 | 4.26 (16/376) | 0.77 (1/130) | 0.0856 |  | 0 (0/376) | 0 (0/130) | 1 | |
|  | 12 | 1.86 (7/376) | 0 (0/130) | 0.1990 |  | 0 (0/376) | 0 (0/130) | 1 | |
|  | 13 | 0 (0/376) | 0.77 (1/130) | 0.2569 |  | 6.12 (23/376) | 4.62 (6/130) | 0.6634 | |
|  | 14 | 5.59 (21/376) | 2.31 (3/130) | 0.1555 |  | 0 (0/376) | 0 (0/130) | 1 | |
|  | 15 | 0 (0/376) | 0 (0/130) | 1 |  | 1.86 (7/376) | 0 (0/130) | 0.1990 | |
|  | 16 | 0 (0/376) | 0 (0/130) | 1 |  | 0.53 (2/376) | 4.62 (6/130) | 0.0046 | |
|  | 17 | 0 (0/376) | 0 (0/130) | 1 |  | 0.80 (3/376) | 5.38 (7/130) | 0.0038 | |
|  | 18 | 1.86 (7/376) | 0.77 (1/130) | 0.6864 |  | 2.39 (9/376) | 0.77 (1/130) | 0.4648 | |
|  | 19 | 3.46 (13/376) | 0 (0/130) | 0.0261 |  | 0 (0/376) | 0 (0/130) | 1 | |
|  | 20 | 6.38 (24/376) | 6.15 (8/130) | 1 |  | 0 (0/376) | 0 (0/130) | 1 | |
|  | 21 | 2.39 (9/376) | 4.62 (6/130) | 0.2298 |  | 0 (0/376) | 0.77 (1/130) | 0.2569 | |
|  | 22 | 2.66 (10/376) | 0 (0/130) | 0.0712 |  | 0 (0/376) | 0 (0/130) | 1 | |
|  | 23 | 15.16 (57/376) | 8.46 (11/130) | 0.0539 |  | 9.57 (36/376) | 16.15 (21/130) | 0.0526 | |
| **Over-five**  **(≥5 years)** | 1 | 4.72 (5/106) | 0 (0/48) | 0.3253 |  | 0 (0/106) | 0 (0/48) | 1 | |
|  | 2 | 0.94 (1/106) | 0 (0/48) | 1 |  | 2.83 (3/106) | 8.33 (4/48) | 0.2051 | |
|  | 3 | 0 (0/106) | 0 (0/48) | 1 |  | 3.77 (4/106) | 2.08 (1/48) | 1 | |
|  | 4 | 0 (0/106) | 0 (0/48) | 1 |  | 2.83 (3/106) | 2.08 (1/48) | 1 | |
|  | 5 | 0 (0/106) | 0 (0/48) | 1 |  | 4.72 (5/106) | 4.17 (2/48) | 1 | |
|  | 6 | 0 (0/106) | 0 (0/48) | 1 |  | 0.94 (1/106) | 0 (0/48) | 1 | |
|  | 7 | 1.89 (2/106) | 0 (0/48) | 1 |  | 0.94 (1/106) | 8.33 (4/48) | 0.0330 | |
|  | 8 | 0.94 (1/106) | 2.08 (1/48) | 0.5276 |  | 0 (0/106) | 0 (0/48) | 1 | |
|  | 9 | 0 (0/106) | 0 (0/48) | 1 |  | 5.66 (6/106) | 8.33 (4/48) | 0.5035 | |
|  | 10 | 3.77 (4/106) | 2.08 (1/48) | 1 |  | 0.94 (1/106) | 0 (0/48) | 1 | |
|  | 11 | 3.77 (4/106) | 0 (0/48) | 0.3105 |  | 0 (0/106) | 0 (0/48) | 1 | |
|  | 12 | 2.83 (3/106) | 2.08 (1/48) | 1 |  | 0 (0/106) | 0 (0/48) | 1 | |
|  | 13 | 0 (0/106) | 2.08 (1/48) | 0.3117 |  | 8.49 (9/106) | 2.08 (1/48) | 0.1740 | |
|  | 14 | 5.66 (6/106) | 0 (0/48) | 0.1777 |  | 0 (0/106) | 0 (0/48) | 1 | |
|  | 15 | 0 (0/106) | 0 (0/48) | 1 |  | 5.66 (6/106) | 2.08 (1/48) | 0.4358 | |
|  | 16 | 0 (0/106) | 0 (0/48) | 1 |  | 3.77 (4/106) | 0 (0/48) | 0.3105 | |
|  | 17 | 0 (0/106) | 4.17 (2/48) | 0.0957 |  | 2.83 (3/106) | 6.25 (3/48) | 0.3764 | |
|  | 18 | 1.89 (2/106) | 0 (0/48) | 1 |  | 2.83 (3/106) | 2.08 (1/48) | 1 | |
|  | 19 | 0 (0/106) | 4.17 (2/48) | 0.0957 |  | 0 (0/106) | 0 (0/48) | 1 | |
|  | 20 | 0.94 (1/106) | 6.25 (3/48) | 0.0900 |  | 0 (0/106) | 0 (0/48) | 1 | |
|  | 21 | 2.83 (3/106) | 2.08 (1/48) | 1 |  | 0 (0/106) | 2.08 (1/48) | 0.3117 | |
|  | 22 | 1.89 (2/106) | 4.17 (2/48) | 0.5890 |  | 0 (0/106) | 0 (0/48) | 1 | |
|  | 23 | 10.38 (11/106) | 2.08 (1/48) | 0.1055 |  | 11.32 (12/106) | 18.75 (9/48) | 0.2165 | |
| *P-values were calculated using the Fisher’s Exact Test. | | | | | | | | | |

**Supplementary Table 3. The odds ratio for carriage of genomic clusters (GC) in under-fives relative to over-fives pre- and post-vaccination.**

| **Genomic cluster (GC)** | **Under-five** | **Over-five** | **Vaccination period** | **Odds ratio**  **(95% CI)** | **Under-five** | **Over-five** | **Vaccination period** | **Odds ratio**  **(95% CI)** |
| --- | --- | --- | --- | --- | --- | --- | --- | --- |
| GC1 | 14 | 5 | Pre-PCV | 0.77 (0.47,1.26) | 4 | 3 | Post-PCV | 0.63 (0.31,1.25) |
| GC2 | 18 | 4 | Pre-PCV | 1.19 (0.71,2.00) | 8 | 4 | Post-PCV | 0.92 (0.51,1.64) |
| GC3 | 12 | 4 | Pre-PCV | 0.80 (0.47,1.38) | 10 | 5 | Post-PCV | 0.93 (0.55,1.59) |
| GC4 | 5 | 3 | Pre-PCV | 0.46 (0.24,0.88) | 5 | 1 | Post-PCV | 1.57 (0.68,3.60) |
| GC5 | 8 | 5 | Pre-PCV | 0.45 (0.27,0.78) | 6 | 2 | Post-PCV | 1.21 (0.59,2.45) |
| GC6 | 11 | 1 | Pre-PCV | 1.90 (0.88,4.11) | 2 | 0 | Post-PCV | 1.55 (0.48,4.98) |
| GC7 | 13 | 4 | Pre-PCV | 0.87 (0.51,1.48) | 10 | 4 | Post-PCV | 1.14 (0.65,2.00) |
| GC8 | 8 | 1 | Pre-PCV | 1.41 (0.64,3.11) | 3 | 0 | Post-PCV | 2.09 (0.67,6.46) |
| GC9 | 14 | 6 | Pre-PCV | 0.65 (0.41,1.05) | 2 | 5 | Post-PCV | 0.24 (0.11,0.49) |
| GC10 | 27 | 6 | Pre-PCV | 1.27 (0.82,1.96) | 2 | 0 | Post-PCV | 1.55 (0.48,4.98) |
| GC11 | 16 | 4 | Pre-PCV | 1.06 (0.63,1.79) | 1 | 1 | Post-PCV | 0.50 (0.18,1.39) |
| GC12 | 6 | 4 | Pre-PCV | 0.43 (0.23,0.77) | 0 | 0 | Post-PCV | 0.51 (0.12,2.11) |
| GC13 | 23 | 9 | Pre-PCV | 0.73 (0.49,1.08) | 7 | 2 | Post-PCV | 1.39 (0.69,2.80) |
| GC14 | 21 | 6 | Pre-PCV | 0.98 (0.63,1.53) | 2 | 2 | Post-PCV | 0.50 (0.22,1.15) |
| GC15 | 7 | 6 | Pre-PCV | 0.34 (0.20,0.58) | 0 | 1 | Post-PCV | 0.25 (0.07,0.86) |
| GC16 | 2 | 4 | Pre-PCV | 0.18 (0.09,0.38) | 5 | 1 | Post-PCV | 1.57 (0.68,3.60) |
| GC17 | 3 | 3 | Pre-PCV | 0.30 (0.15,0.62) | 6 | 4 | Post-PCV | 0.70 (0.38,1.29) |
| GC18 | 14 | 7 | Pre-PCV | 0.57 (0.36,0.89) | 2 | 3 | Post-PCV | 0.37 (0.17,0.80) |
| GC19 | 13 | 0 | Pre-PCV | 4.50 (1.59,12.73) | 0 | 0 | Post-PCV | 0.51 (0.12,2.11) |
| GC20 | 24 | 1 | Pre-PCV | 4.11 (1.95,8.63) | 6 | 4 | Post-PCV | 0.70 (0.38,1.29) |
| GC21 | 9 | 3 | Pre-PCV | 0.77 (0.42,1.41) | 7 | 4 | Post-PCV | 0.81 (0.45,1.46) |
| GC22 | 10 | 2 | Pre-PCV | 1.15 (0.59,2.22) | 0 | 1 | Post-PCV | 0.25 (0.07,0.86) |
| GC23 | 90 | 26 | Pre-PCV | 1.07 (0.83,1.37) | 30 | 13 | Post-PCV | 1.18 (0.82,1.71) |

**Supplementary Table 4. Frequency of carried pneumococcal serotypes in pre- and post-vaccination.**

| **Serotype** | **Frequency in under-fives (%)** | | **Odds ratio** | **P-Value*** | **Frequency in over-fives**  **(%)** | | **Odds ratio** | **P-Value*** |
| --- | --- | --- | --- | --- | --- | --- | --- | --- |
|  | **Pre-vaccination** | **Post-vaccination** |  |  | **Pre-vaccination** | **Post-vaccination** |  |  |
| 1 | 1.86 (7/376) | 0 (0/130) | 0.3531 | 0.1990 | 0 (0/106) | 0 (0/48) | 2.18 | 1 |
| 10A | 1.06 (4/376) | 0 (0/130) | 0.5695 | 0.5767 | 0 (0/106) | 0 (0/48) | 2.18 | 1 |
| 10B | 2.39 (9/376) | 5.38 (7/130) | 2.3742 | 0.1404 | 3.77 (4/106) | 2.08 (1/48) | 0.86 | 1 |
| 11A | 1.60 (6/376) | 4.62 (6/130) | 2.9680 | 0.0862 | 0.94 (1/106) | 2.08 (1/48) | 2.21 | 0.5276 |
| 12B | 0.27 (1/376) | 0 (0/130) | 1.4351 | 1 | 0 (0/106) | 0 (0/48) | 2.18 | 1 |
| 12F | 0 (0/376) | 0.77 (1/130) | 5.8000 | 0.2569 | 0 (0/106) | 2.08 (1/48) | 4.46 | 0.3117 |
| 13 | 3.72 (14/376) | 1.54 (2/130) | 0.5628 | 0.3812 | 5.66 (6/106) | 8.33 (4/48) | 1.60 | 0.5035 |
| 14 | 2.66 (10/376) | 2.31 (3/130) | 1.0426 | 1 | 4.72 (5/106) | 0 (0/48) | 0.35 | 0.3253 |
| 15A | 1.06 (4/376) | 2.31 (3/130) | 2.3313 | 0.3804 | 0 (0/106) | 4.17 (2/48) | 6.83 | 0.0957 |
| 15B/C | 2.66 (10/376) | 9.23 (12/130) | 3.6448 | 0.0042 | 5.66 (6/106) | 2.08 (1/48) | 0.60 | 0.4358 |
| 16F | 6.91 (26/376) | 6.15 (8/130) | 0.9512 | 0.8419 | 9.43 (10/106) | 4.17 (2/48) | 0.56 | 0.3431 |
| 17F | 0 (0/376) | 0 (0/130) | 2.8779 | 1 | 0.94 (1/106) | 0 (0/48) | 1.08 | 1 |
| 18A | 1.33 (5/376) | 0.77 (1/130) | 0.9538 | 1 | 0.94 (1/106) | 0 (0/48) | 1.08 | 1 |
| 18C | 3.46 (13/376) | 0.77 (1/130) | 0.4000 | 0.1300 | 2.83 (3/106) | 4.17 (2/48) | 1.66 | 0.6473 |
| 19A | 4.79 (18/376) | 3.08 (4/130) | 0.7439 | 0.6176 | 4.72 (5/106) | 6.25 (3/48) | 1.48 | 0.7050 |
| 19B | 1.33 (5/376) | 3.85 (5/130) | 2.9524 | 0.1341 | 2.83 (3/106) | 2.08 (1/48) | 1.08 | 1 |
| 19F | 9.57 (36/376) | 6.92 (9/130) | 0.7554 | 0.4746 | 0.94 (1/106) | 6.25 (3/48) | 4.61 | 0.0900 |
| 2 | 0 (0/376) | 0 (0/130) | 2.8779 | 1 | 0.94 (1/106) | 0 (0/48) | 1.08 | 1 |
| 20 | 0.27 (1/376) | 1.54 (2/130) | 4.3721 | 0.1635 | 0 (0/106) | 2.08 (1/48) | 4.46 | 0.3117 |
| 21 | 3.72 (14/376) | 3.08 (4/130) | 0.9528 | 1 | 0.94 (1/106) | 2.08 (1/48) | 2.21 | 0.5276 |
| 22A | 0.53 (2/376) | 0.77 (1/130) | 1.9231 | 1 | 1.89 (2/106) | 0 (0/48) | 0.71 | 1 |
| 23A | 0 (0/376) | 2.31 (3/130) | 11.7813 | 0.0167 | 0.94 (1/106) | 0 (0/48) | 1.08 | 1 |
| 23B | 0.80 (3/376) | 1.54 (2/130) | 2.1744 | 0.6068 | 0.94 (1/106) | 0 (0/48) | 1.08 | 1 |
| 23F | 6.65 (25/376) | 2.31 (3/130) | 0.4231 | 0.0744 | 5.66 (6/106) | 2.08 (1/48) | 0.60 | 0.4358 |
| 24 | 0.27 (1/376) | 0 (0/130) | 1.4351 | 1 | 0 (0/106) | 0 (0/48) | 2.18 | 1 |
| 28F | 0 (0/376) | 5.38 (7/130) | 24.3226 | 0.0001 | 0 (0/106) | 6.25 (3/48) | 9.30 | 0.0290 |
| 29 | 0.27 (1/376) | 0 (0/130) | 1.4351 | 1 | 1.89 (2/106) | 2.08 (1/48) | 1.46 | 1 |
| 3 | 2.39 (9/376) | 1.54 (2/130) | 0.8558 | 0.7369 | 2.83 (3/106) | 4.17 (2/48) | 1.66 | 0.6473 |
| 31 | 1.06 (4/376) | 0.77 (1/130) | 1.1477 | 1 | 2.83 (3/106) | 2.08 (1/48) | 1.08 | 1 |
| 33D | 0.53 (2/376) | 2.31 (3/130) | 3.9063 | 0.1099 | 0 (0/106) | 0 (0/48) | 2.18 | 1 |
| 34 | 2.93 (11/376) | 1.54 (2/130) | 0.7093 | 0.5303 | 3.77 (4/106) | 4.17 (2/48) | 1.31 | 1 |
| 35A | 0.27 (1/376) | 0.77 (1/130) | 2.8923 | 0.4482 | 0 (0/106) | 0 (0/48) | 2.18 | 1 |
| 35B | 1.60 (6/376) | 1.54 (2/130) | 1.2326 | 1 | 2.83 (3/106) | 2.08 (1/48) | 1.08 | 1 |
| 35F | 0.27 (1/376) | 1.54 (2/130) | 4.3721 | 0.1635 | 0 (0/106) | 0 (0/48) | 2.18 | 1 |
| 38 | 0 (0/376) | 0 (0/130) | 2.8779 | 1 | 0 (0/106) | 2.08 (1/48) | 4.46 | 0.3117 |
| 4 | 1.60 (6/376) | 0.77 (1/130) | 0.8154 | 0.6837 | 3.77 (4/106) | 2.08 (1/48) | 0.86 | 1 |
| 42 | 0.27 (1/376) | 0 (0/130) | 1.4351 | 1 | 0 (0/106) | 0 (0/48) | 2.18 | 1 |
| 5 | 2.66 (10/376) | 0 (0/130) | 0.2547 | 0.0712 | 1.89 (2/106) | 2.08 (1/48) | 1.46 | 1 |
| 6A | 8.51 (32/376) | 5.38 (7/130) | 0.6745 | 0.3398 | 6.60 (7/106) | 0 (0/48) | 0.26 | 0.0994 |
| 6B | 11.17 (42/376) | 5.38 (7/130) | 0.5026 | 0.0589 | 2.83 (3/106) | 0 (0/48) | 0.53 | 0.5525 |
| 7C | 0.80 (3/376) | 6.15 (8/130) | 6.8415 | 0.0013 | 2.83 (3/106) | 8.33 (4/48) | 2.89 | 0.2051 |
| 7F | 0.80 (3/376) | 0 (0/130) | 0.7137 | 0.5730 | 0.94 (1/106) | 0 (0/48) | 1.08 | 1 |
| 9L | 0.80 (3/376) | 0.77 (1/130) | 1.4385 | 1 | 0 (0/106) | 2.08 (1/48) | 4.46 | 0.3117 |
| 9V | 4.79 (18/376) | 4.62 (6/130) | 1.0581 | 1 | 4.72 (5/106) | 6.25 (3/48) | 1.48 | 0.7050 |
| NT | 2.39 (9/376) | 2.31 (3/130) | 1.1500 | 1 | 7.55 (8/106) | 6.25 (3/48) | 0.96 | 1 |
| *P-values were calculated using the Fisher’s Exact Test. | | | | | | | | |

**Supplementary Table 5. Proportion of vaccine type (VT) and non-vaccine type (NVT) pneumococcal carried strains in pre- and post-vaccination.**

| **Vaccine status** | **Frequency in**  **under-fives (%)** | | ***P*-value*** | **Frequency in**  **over-fives (%)** | | ***P*-value*** |
| --- | --- | --- | --- | --- | --- | --- |
|  | **Pre-vaccination** | **Post-vaccination** |  | **Pre-vaccination** | **Post-vaccination** |  |
| **Vaccine type (VT)** | 60.90 (229/376) | 33.08 (43/130) | 4.80×10^-08^ | 42.45 (45/106) | 33.33 (16/48) | 0.3739 |
| **Non-vaccine type (NVT)** | 39.10 (147/376) | 66.92 (87/130) |  | 57.55 (61/106) | 66.67 (32/48) |  |
| **P*-value for difference in frequency of VT and NVT pre- and post-vaccination calculated using Fisher’s Exact Test. | | | | | | |

**Supplementary Table 6. The Simpson diversity index for composition of serotypes and STs in pneumococcal carriers before and after vaccination.**

| **Strain type** | **Age category** | **Category** | **Pre-PCV** | | | **Post-PCV** | | | ***P*-value*** |
| --- | --- | --- | --- | --- | --- | --- | --- | --- | --- |
|  |  |  | **Value** | **95%**  **lower CI** | **95%**  **upper CI** | **Value** | **95%**  **lower CI** | **95%**  **upper CI** |  |
| **Serotype** | Under-five | VT | 0.928 | 0.91 | 0.946 | 0.883 | 0.849 | 0.917 | 0.022 |
|  |  | NVT | 0.933 | 0.857 | 1 | 0.908 | 0.86 | 0.957 | 0.542 |
|  |  | All | 0.951 | 0.937 | 0.964 | 0.962 | 0.954 | 0.97 | 0.301 |
|  | Over-five | VT | 0.922 | 0.899 | 0.944 | 0.943 | 0.927 | 0.96 | 0.132 |
|  |  | NVT | 0.944 | 0.912 | 0.975 | 0.958 | 0.931 | 0.984 | 0.521 |
|  |  | All | 0.961 | 0.944 | 0.978 | 0.972 | 0.959 | 0.985 | 0.301 |
| **Sequence type** | Under-five | VT | 0.971 | 0.956 | 0.986 | 0.975 | 0.961 | 0.988 | 0.776 |
|  |  | NVT | 0.983 | 0.956 | 1 | 0.958 | 0.91 | 1 | 0.465 |
|  |  | All | 0.989 | 0.985 | 0.993 | 0.987 | 0.982 | 0.991 | 0.421 |
|  | Over-five | VT | 0.965 | 0.95 | 0.98 | 0.979 | 0.97 | 0.988 | 0.097 |
|  |  | NVT | 0.972 | 0.941 | 1 | 0.98 | 0.956 | 1 | 0.694 |
|  |  | All | 0.986 | 0.977 | 0.995 | 0.982 | 0.967 | 0.998 | 0.713 |
| **P*-value for differences in the Gini-Simpson diversity index (D) between classifications of strains pre- and post-introduction of PCV13 were calculated by resampling. The Jackknife pseudo-values confidence intervals (CI) from resampling are not show because these were nearly identical to the ‘normal’ CI values shown in the table and the non-approximated CIs (CINA) not shown in the table. The *P*-values were calculated from matched datasets of the pre- and post-vaccination datasets whereby the larger of the two datasets was randomly sub-sampling to equal the size of the smaller dataset. | | | | | | | | | |

**Supplementary Table 7. The Simpson diversity index (D) between VT and NVT serotypes in pneumococcal carriers before and after vaccination.**

| **Strain type** | **Age category** | **Sampling period** | **VT** | | | | **NVT** | | | | ***P*-value*** |
| --- | --- | --- | --- | --- | --- | --- | --- | --- | --- | --- | --- |
|  |  |  | **Value** | **95% lower CI** | **95% upper CI** | **Value** | | **95% lower CI** | **95% upper CI** |  | |
| **Serotype** | Under-five | Pre-PCV | 0.928 | 0.91 | 0.946 | 0.933 | | 0.857 | 1 | 0.637 | |
|  |  | Post-PCV | 0.883 | 0.849 | 0.917 | 0.908 | | 0.86 | 0.957 | 0.004 | |
|  | Over-five | Pre-PCV | 0.922 | 0.899 | 0.944 | 0.944 | | 0.912 | 0.975 | 0.6 | |
|  |  | Post-PCV | 0.943 | 0.927 | 0.96 | 0.958 | | 0.931 | 0.984 | 0.508 | |
| **Sequence type** | Under-five | Pre-PCV | 0.971 | 0.956 | 0.986 | 0.983 | | 0.956 | 1 | 0.876 | |
|  |  | Post-PCV | 0.965 | 0.95 | 0.98 | 0.972 | | 0.941 | 1 | 0.053 | |
|  | Over-five | Pre-PCV | 0.975 | 0.961 | 0.988 | 0.958 | | 0.91 | 1 | 0.706 | |
|  |  | Post-PCV | 0.979 | 0.97 | 0.988 | 0.98 | | 0.956 | 1 | 0.297 | |
| **P*-value for differences in the Gini-Simpson diversity index (D) between classifications of strains pre- and post-introduction of PCV13 were calculated by resampling. The Jackknife pseudo-values confidence intervals (CI) from resampling are not show because these were nearly identical to the ‘normal’ CI values shown in the table and the non-approximated CIs (CINA) not shown in the table. The *P*-values were calculated from matched datasets of the pre- and post-vaccination datasets whereby the larger of the two datasets was randomly sub-sampling to equal the size of the smaller dataset. | | | | | | | | | | | |

**Supplementary Table 8. Frequency of antibiotic resistant pneumococcal strains in nasopharyngeal carriage pre- and post-vaccination.**

| **Antibiotic** | **Antibiotic resistance gene** | **Highly associated**  **serotypes** | **Highly associated**  **GCs** | **Genotypic resistance rate (%)** | | **P-value*** |
| --- | --- | --- | --- | --- | --- | --- |
|  |  |  |  | **Pre-vaccination** | **Post-vaccination** |  |
| **Chloramphenicol** | *cat*(_pC194_) | 1,12F,6B | 8,23 | 2.70 (13/482) | 2.81 (5/178) | 1 |
| **Tetracycline** | *tet*(M) | 14,10A/B,6B,3,23F,19A/F,1,12F | 1,3,8,12,14,20,23 | 23.24 (112/482) | 20.22 (36/178) | 0.4621 |
| **Erythromycin** | *erm*(B) | 23F | 14 | 1.04 (5/482) | 2.25 (4/178) | 0.261 |
|  | *mef*(A) | 15B/C,6A,19F | 5,14,20 | 1.24 (6/482) | 0 (0/178) | 1 |
|  | *mef*(E) | 13,23F,35A | 9,14,23 | 2.07 (10/482) | 2.25 (4/178) | 1 |
|  | Combined | 15B/C,6A,13,19F,23F | 5,9,14,20,23 | 4.15 (20/482) | 5.62 (10/178) | 0.406 |
| **Penicillin** | *pbp1a/2b/2x*^§^ | Multiple | Multiple | 31.86 (151/474) ^§§^ | 32.58 (58/178) ^§§^ | 0.8512 |
| *P-values were calculated using the Fisher’s Exact Test.  ^§^Penicillin resistance was determined by the specific alleles associated with resistance.  ^§§^Eight strains were not included. no genotypic resistance data (5), *pbp* sequences unavailable (3). | | | | | | |

**Supplementary Table 9. Coefficients for the effect of sampling period on presence and absence of accessory genes using logistic regression accounting for age and serotype category after Bonferroni adjustment for multiple testing.**

| **Accessory gene*** | **Odds ratio (95% CI)** | **Unadjusted *P-*value** | **Adjusted *P-*value** | **Description/product** |
| --- | --- | --- | --- | --- |
| COG_6818 | 3.34 (2.24,4.99) | 3.75×10^-09^ | 9.71×10^-06^ | Glycosyl transferase |
| COG_7104 | 3.34 (2.20,5.06) | 1.33×10^-08^ | 3.46×10^-05^ | Double glycine cleavage site bacteriolysin superfamily |
| *hsdR_1* | 3.34 (2.20,5.06) | 1.33×10^-08^ | 3.46×10^-05^ | Type I restriction-modification system, R subunit |
| COG_2038 | 2.78 (1.91,4.06) | 1.12×10^-07^ | 2.91×10^-04^ | ABC transporter permease |
| COG_900 | 2.78 (1.91,4.06) | 1.12×10^-07^ | 2.91×10^-04^ | IS630-Spn1, transposase Orf1 |
| COG_5542 | 2.84 (1.93,4.18) | 1.17×10^-07^ | 3.03×10^-04^ | Replication protein |
| *ptrB* | 0.30 (0.19,0.47) | 3.48×10^-07^ | 9.02×10^-04^ | Prolyl oligopeptidase family protein |
| COG_4000 | 2.95 (1.94,4.48) | 3.96×10^-07^ | 1.03×10^-03^ | gp27 phage protein |
| *rlmN* | 2.95 (1.94,4.48) | 3.96×10^-07^ | 1.03×10^-03^ | Ribosomal RNA large subunit methyltransferase N |
| COG_4863 | 3.34 (2.09,5.33) | 4.59×10^-07^ | 1.19×10^-03^ | Hypothetical protein |
| COG_5488 | 2.54 (1.76,3.65) | 5.24×10^-07^ | 1.36×10^-03^ | Phage protein |
| COG_842 | 0.40 (0.28,0.58) | 6.98×10^-07^ | 1.81×10^-03^ | ABC transporter permease |
| IS861 truncation | 0.40 (0.28,0.58) | 6.98×10^-07^ | 1.81×10^-03^ | IS861 truncation |
| COG_1881 | 2.74 (1.82,4.14) | 1.59×10^-06^ | 4.11×10^-03^ | SNF2 family protein |
| COG_802 | 2.74 (1.82,4.14) | 1.59×10^-06^ | 4.11×10^-03^ | Transposase |
| COG_4974 | 3.00 (1.91,4.72) | 1.93×10^-06^ | 5.00×10^-03^ | Membrane associated protein |
| COG_1144 | 2.41 (1.67,3.50) | 3.15×10^-06^ | 8.17×10^-03^ | 2-isopropylmalate synthase |
| COG_3978 | 0.41 (0.28,0.60) | 4.17×10^-06^ | 1.08×10^-02^ | Rep protein |
| COG_5509 | 0.41 (0.28,0.60) | 4.17×10^-06^ | 1.08×10^-02^ | Phage protein gp27 |
| COG_6116 | 0.41 (0.28,0.60) | 4.17×10^-06^ | 1.08×10^-02^ | ABC transporter permease |
| *saeS* | 0.41 (0.28,0.60) | 4.21×10^-06^ | 1.09×10^-02^ | Histidine kinase |
| COG_6305 | 0.41 (0.28,0.60) | 4.77×10^-06^ | 1.24×10^-02^ | Bacteriocin |
| COG_748 | 0.41 (0.28,0.60) | 4.77×10^-06^ | 1.24×10^-02^ | Hypothetical protein |
| *blpPQ* | 0.19 (0.09,0.39) | 5.20×10^-06^ | 1.35×10^-02^ | Bacteriocin BlpPQ |
| COG_2175 | 0.41 (0.28,0.61) | 5.84×10^-06^ | 1.51×10^-02^ | Hypothetical protein |
| COG_2726 | 0.41 (0.28,0.61) | 5.84×10^-06^ | 1.51×10^-02^ | Tn5252, Orf 9 protein |
| COG_3239 | 0.41 (0.28,0.61) | 5.84×10^-06^ | 1.51×10^-02^ | Type II restriction endonuclease DpnI |
| *celB* | 2.30 (1.60,3.31) | 6.87×10^-06^ | 1.78×10^-02^ | Competence protein CelB |
| *yetF* | 2.60 (1.71,3.96) | 7.29×10^-06^ | 1.89×10^-02^ | Putative membrane protein yetF |
| COG_40 | 2.34 (1.60,3.41) | 1.04×10^-05^ | 2.69×10^-02^ | Transposase |
| *comA* | 0.44 (0.30,0.63) | 1.50×10^-05^ | 3.88×10^-02^ | Competence factor transporting ATP-binding protein/permease ComA |
| COG_2501 | 0.44 (0.30,0.63) | 1.50×10^-05^ | 3.88×10^-02^ | Acetyltransferase |
| COG_66 | 0.44 (0.30,0.63) | 1.50×10^-05^ | 3.88×10^-02^ | Transposase |
| *ltrA* | 0.44 (0.30,0.63) | 1.50×10^-05^ | 3.88×10^-02^ | Reverse transcriptase/maturase, group FT II introns |
| COG_5490 | 2.29 (1.57,3.33) | 1.58×10^-05^ | 4.10×10^-02^ | Diadenosine tetraphosphate |
| *manY_2* | 2.29 (1.57,3.33) | 1.58×10^-05^ | 4.10×10^-02^ | PTS system mannose/fructose/N-acetylgalactosamine-specific transporter subunit IIC |
| COG_1331 | 2.87 (1.78,4.64) | 1.61×10^-05^ | 4.18×10^-02^ | Phage protein |
| COG_3738 | 2.87 (1.78,4.64) | 1.61×10^-05^ | 4.18×10^-02^ | Phage-like protein |
| COG_6858 | 2.87 (1.78,4.64) | 1.61×10^-05^ | 4.18×10^-02^ | FIG01115102: hypothetical protein |
| COG_6937 | 0.43 (0.29,0.63) | 1.64×10^-05^ | 4.24×10^-02^ | Capsule biosynthesis repeating unit flippase Wzx |
| COG_1582 | 0.40 (0.26,0.61) | 1.86×10^-05^ | 4.83×10^-02^ | FIG01114725: hypothetical protein |
| COG_1890 | 0.40 (0.26,0.61) | 1.86×10^-05^ | 4.83×10^-02^ | ABC transporter ATP-binding protein |
| *COG cluster of orthologous genes | | | | |
